# “Good childhood”: children’s perception and evaluation of transnational families

**DOI:** 10.3389/fsoc.2024.1470541

**Published:** 2024-12-05

**Authors:** Alexandra König, Jessica Schwittek, Katarzyna Jendrzey

**Affiliations:** University of Duisburg-Essen, Duisburg, Germany

**Keywords:** children’s perspective, good childhood, migrating parents, parental presenteeism, mobility within the EU, migration Poland, normativity, transnational families

## Abstract

In public debates, transnational families are portrayed as a deviation from the norm of “good childhood.” In Europe, this is emphasized by the term “Euro-orphans,” branding parents’ (especially mothers’) absence as a violation and scandalizing it. Children’s voices are rarely heard in public discourse, and although research is now turning its attention to the “stayer children,” they and their perspectives on transnational family life remain underrepresented, especially in Europe. In a German-Polish project, we investigate how children perceive and evaluate transnational family life based on 27 group discussions with 12-14-year-olds (with and without own transnational family experience) in Poland. The analysis shows that (1) the presence of parents is central to the normative pattern of a good childhood from children’s perspective, but (2) they use differentiated criteria when assessing (temporary) parental migration, i.e., they do not refer to “universal” needs of children. Additionally, (3) children request that they be informed about the migration-decision early on and involved in the organization of the time of separation to make it as acceptable as possible for them. Thereby, they offer interpretations of transnational families that contribute to erode the norm of good childhood. We see our paper as a sociologically and socio-politically relevant contribution to expanding the discussion on transnational families, both from the perspective of children who discuss and problematize transnational family life as more than merely a question of violating the norm of parents’ presence, and with our focus on the European region, which provides specific contextual conditions for transnational families.

## 1 Introduction

In the summer of 2023, we three sociologists traveled to a small Polish town on the eastern border. We were warmly welcomed by teachers at the local elementary school. Contact with the school had been established roughly a year earlier when, as part of a German-Polish research project, one of our team was a guest at the school for a few days and interviewed pupils about their views on transnational family life. One year later, the entire project team returned to thank the teachers for their warm and patient support during the data collection, and to talk about transnational families again. We briefly reiterated our interest in transnational families and asked the group whether this family constellation was common there, to which they unanimously answered in the affirmative: Yes, some of the children were “Euro-orphans.” When asked to describe their experiences and perceptions (without mentioning names), one teacher recalled a girl who was suicidal, presumably due to the physical absence of one or both of her parents. With this example—as with the teachers’ use of the term “Euro-orphan”—the conversation begins by referencing the inappropriateness, even dangerousness, of the transnational family arrangement. When we later asked whether transnational family life is often followed by such dramatic consequences for children, a teacher (perhaps the same one) referenced the girl’s brother, who from that teacher’s view stood out positively rather than negatively, with his focused and enthusiastic interest in agriculture—an interest that likely developed thanks to his new care arrangement at his uncle’s.

We recount this episode to show how the teachers’ everyday interpretations could be reminiscent of current public discourses, drawing from the same normative ideal of a family in which both parents are present. The sometimes one-sided discursive linking of the transnational family with the suffering and despair of the children left behind and labeled as “Euro-orphans” (Trusz and Kwiecień, 2012) is exemplary of the “moral panic” that characterizes the discourses on transnational families (Urbańska, 2015). The discourses refer to a specific and narrow norm of good parenting that is closely tied to “presenteeism” (Edgley, 2021). In Poland, this norm of parents’ physical presence in the same place as their children is clearly a powerful one—as in many other countries. The dominance of the norm of parental co-presence has been well established, particularly in the Polish media discourse (Slany et al., 2014), purportedly out of concern for children’s needs and welfare. What is problematic about this—and is the starting point of our contribution—is that this discourse is highly adultist, in the sense that it is determined by adults’ interpretations of the effects of transnational family life on children. We problematize this dominance of adult perspectives against the background of the participatory demand to hear the voice of those affected—in this case the children left behind—rather than (only) talking about them (Prout and Hallett, 2003). Sociological childhood research has methodological approaches and tried and tested instruments at its disposal to (critically) reconstruct the perspective of children (Punch, 2002; Harcourt and Einarsdottir, 2011; for a recent overview on methodological approaches in childhood research, see Spiteri, 2024), from which we draw in this paper. Such reconstruction of children’s perspective makes it possible to feed their voice into the (public) discourse, as a political concern. The second aspect guiding this article is a scientific one: to better understand the complex empirical phenomenon of transnational family life as an increasingly relevant part of the social world. As children occupy specific positions in the social order, especially with regard to its generational structure (Alanen, 2005), their perspectives offer insights into the social world that differ from those of adults. Against the background of these two aspects, we ask in our study how children themselves perceive transnational family arrangements and how they evaluate them—regardless of whether they are growing up in a transnational family themselves. The analysis of group discussions with children aged 12–14 allows us to make a more fundamental contribution to the research in childhood studies on definitions of good childhood (Bühler-Niederberger, 2024) and the construction of the generational order. By reconstructing children’s ideas of a good childhood and their understanding of how both adults and children contribute to it (or fail to do so), our study contributes to overarching sociological questions about the reproduction and negotiation of normative ideas of childhood and family and explores what position children themselves hold.

We explore these questions in an interdisciplinary German-Polish research project called DoDzi^1^ that took us, among other places, to the small elementary school in eastern Poland. In our project, we define transnational families as core families in which fathers and/or mothers (temporarily) migrate, “yet hold together and create ‘familyhood’ in their ability to reconstruct and redefine themselves through time, space, emotions, and materialities” (Walczak, 2016, p. 29ff.; Cienfuegos et al., 2023, p. 3).

The sociological research part of the project focuses on children’s own perceptions and evaluations of this transnational family form. Accordingly, we utilized group discussions to encourage children to talk about the phenomenon. In the following piece, we will summarize the results of our analysis of the group discussions. First, we will outline in section 2 how transnational families are structured in Poland and examine studies that deal with the evaluation of such family arrangements. In section 3, we will then explain our own approach, on which the presentation of results in section 4 is based. Using empirical evidence, we will show how children position themselves in relation to the norm of parental presence and which problems they identify in transnational family arrangements. From this, we will derive which idea of a good childhood is fundamental from children’s perspectives, which will be summarized and discussed in section 5.

## 2 Transnational families—specifics of migration from Poland

Transnational families are not a completely new arrangement in Poland (see Thomas and Znaniecki, 1995), but (temporary) labor migration to neighboring European countries has increased significantly since 2004, with the free movement of workers, and has become a widespread practice (GUS, 2021). An estimated 2 million Polish citizens lived abroad temporarily in 2020, the majority in Germany (GUS, 2023, p. 53; Hut, 2023). Temporary labor migration is a model practiced especially by women, and increasingly also by mothers (Kępińska, 2008; Ryan et al., 2008; on forced migration of women: Urbańska, 2016). For Poland, Walczak (2016) reports that maternal migration has almost doubled since 2008 and amounted to 15% of parental migration in 2014. It can be assumed that roughly every fourth child in Poland had experienced (temporary) transnational family life in the 3 years prior to the study (Walczak, 2008, p. 12; Walczak, 2014, p. 17).^2^ According to teacher estimates, over 6% of their pupils currently live in transnational families, while 20% of pupils self-report doing so (Ostrowska, 2016, p. 15ff.). The discrepancy shown here is already a first indication that this form of family life remains partly invisible. The statistical normality of transnational families stands in contrast to the normative idea of good parenthood, which is primarily characterized by parental presence. In Poland—as in many other countries—labor migration is discussed primarily in terms of parental absence from their children and is frequently scandalized. This can be seen starkly in the term “Euro-orphans,” which emerged in Poland in 2007 (Kawecki and Kwatera, 2015, p. 26) and labels children as parentless. The extent to which the media discourse scandalizes migrating mothers is well documented. Based on an analysis of 500 newspaper articles (including statements by moral entrepreneurs), Urbańska (2015) shows how a moral panic is constructed around the migrating mother (similarly: Walczak, 2016; Lutz, 2018), in which not only the welfare of the child but all of Polish society is threatened. Lutz and Palenga-Möllenbeck (2012, p. 14) describe the logic of medial discourse as “naming, blaming, shaming.”^3^ The migrating mother is stylized as a deviation from the normative pattern of good motherhood not only in media targeting adults, but also in Polish books aimed at young children. Jendrzey (in press) shows for the latter that here, too, the migrating mother is suspicious and either fails in her motherhood or is redeemed at the end of the book only when she decides to return to her children for good (see also: Zając, 2023). The books’ endings are hardly surprising: A “good” mother in Poland is closely linked to the image of a present and family-oriented mother, based on the historical myth of the “Mother-Pole” (Imbierowicz, 2012; Pustułka, 2014; König et al., 2021).

Despite changes in the norm and reality of motherhood, current studies indicate the persistence of the image of the “Mother-Pole” who is permanently and physically present for her family (Kocot-Górecka, 2014, p. 43; ROPS, 2015, p. 51; on the division of responsibilities of Polish couples, see CBOS, 2018; Sikorska, 2019). This expectation of permanent presence was promoted in particular by the PiS government’s family-centered national policy, which is closely linked to the values of the Catholic Church. In this context, Wawrzyniak and Sikorska (2024) speak of a return to familism, which aims to strengthen traditional family models and gender-specific roles while alternative lifestyles are increasingly marginalized.

Within this framework, a migrating mother violates the ideal of a present mother and becomes an object of social interest, as proven not only by media and political discourses but also by debates among religious representatives, who have adopted the topic for themselves and repeatedly discuss it in the context of a threat to the family (Szwed, 2018) and to national continuity (Urbańska, 2015, p. 47). Titkow (2007, p. 70) argues in this context that Catholic representatives have reduced Polish women to the areas of “children, kitchen, church.” This underlines the ambivalent position of the (migrant) mother who, as a breadwinner, assumes a central role in improving the quality of life of her family and at the same time must continue to meet the demands placed on her as the family’s “manager of care and everyday life” (Sikorska, 2019, p. 238). In contrast, migrating fathers are less likely to be the subject of public discourse (and scandalization), as their mobilities correspond to the normative pattern of the “male breadwinner” (Slany et al., 2017), and their break with the norm of parental presenteeism can thus conceivably be relativized or legitimized.

Transnational families are a global phenomenon attracting academic attention in various regions of the world. Research has primarily looked at motherhood from a distance and at the impact of women’s migration on the gender order (Hondagneu-Sotelo and Avila, 1997; Parreñas, 2005). For example, studies show how mothers legitimize their absence as sacrificing for their children (Fresnoza-Flot, 2014) or that the responsibilities of migrating mothers are primarily assumed by other women, maintaining the gender order (Lutz and Palenga-Möllenbeck, 2012, p. 32). Particularly in these early studies, the generational order is not addressed. The focus is not so much on childhood as on motherhood. For some time, family research has also turned its attention to this phenomenon, studying doing family from a distance and how family is created over distance, far beyond remittances. The socio-technical possibilities are being investigated (Greschke and Motowidlo, 2020; Waruwu, 2022) and the contributions of stayer children increasingly considered (Christ, 2017). Children’s agency is identified by their complicity (Nosek-Kozłowska, 2021) and/or resistant practices (Bonizzoni and Leonini, 2013; Hoang et al., 2015). The studies also provide indications of the impositions that arise for children in this arrangement and the limited maneuvering room with which the children are left. Studies in Asian countries provide an explanation for the effectiveness of filial piety for the parent–child arrangement in some regions (Madianou and Miller, 2012; Christ, 2017; Gu, 2022). In these studies, children are conceptualized primarily as family members; their relationships with significant others (for example peers or teachers) are largely neglected. Another line of research focuses on children and examines the effects growing up in such family arrangements has on their objective and subjective well-being. The results are extremely heterogeneous (Antia et al., 2020),^4^ demonstrating how differently transnational family life can be experienced (see also Nosek-Kozłowska, 2021). One finding from research on children’s subjective well-being (SWB) more generally (not only in transnational families) should be highlighted here, as it is of particular relevance to our analysis. Based on data from the Children’s Worlds Survey of 8-, 10-, and 12-year-olds in 18 countries, Lee and Yoo (2017, p. 19) conclude that “*freedom to choose* and *self* are [the] most important factors for determining a country’s overall level of children’s SWB” (emphasis added).

How labor migration is structured, how family life can be and is organized long-distance, and what significance this has for children differ immensely between countries and regions, making research findings hardly comparable or transferable to transnational childhoods in Poland.^5^ In contrast to frequently studied countries such as Mexico or the Philippines, at least four aspects are specific to Poland. Firstly, labor migration within Europe has been legally possible without restrictions for Polish citizens since its EU accession in 2004. Due to the full freedom of movement for workers, migration patterns in Poland have changed: European migration has replaced transatlantic migration (Okólski and Salt, 2014; PAN, 2014; Ślusarczyk, 2014), and both temporary labor migration and “circular migration” have increased (Goździak and Pawlak, 2016, p. 109). Closely related to this is the fact that, secondly, the transnational space (Faist et al., 2014) within Europe is smaller than for many of the other transnational families typically studied. The geographical proximity allows, for example, for reciprocal visits and for alternating work and family modes. Another characteristic of labor migration from Poland is, thirdly, the lower economic disparity between Poland and other EU-countries. Migration is less a matter of existential need and more an alternative for a better financial livelihood. Fourthly, it must be considered that transnational families also differ in terms of the generational and gender-specific orders of their societies of origin. With regard to variations in the generational order, Bühler-Niederberger (2021), for example, distinguishes between societies that are more strongly based on an interdependence model versus an independence model, indicating which intergenerational solidarities are expected. Filial piety, for example, underpins the interdependence model. What is expected of children and what is considered a “good childhood” varies depending on the historical and social context. Qvortrup (2005, p. 31; authors’ translation) summarizes this idea as follows: “It is significant that the architecture of childhood is fundamentally shaped by society—more precisely: by its prevailing parameters—and that childhood is thus to be understood as an integrated element of and within society, which interacts with other generational groups.” While in many countries children of migrant parents are seen as “left behinds,” in others they are not stigmatized in this way. In Ghana, for instance, the subjective well-being of children of migrant parents is even more pronounced because a culture of migration prevails there, and transnational families are an accepted family form (Cebotari et al., 2018). Other studies, such as from Moldova, show how children whose parents migrate try to conceal this and thus engage in stigma management. Last but not least, the aspect of identification with the traditional role model in the household plays an important role. With regard to the gender order, Parreñas’ (2005, p. 97) study is a classic example: She shows that the children in the Philippines with migrating mothers suffer more from migration than the children of migrating fathers, as the conventional division of labor and traditional notions of motherhood are at odds with their new household configurations.

With regard to the Polish context, there are some studies on transnational family arrangements but little research on how children there evaluate the transnational family arrangement. The few available findings indicate that they differentiate their assessment of transnational family life depending, for example, on family relationships prior to migration, the (active) involvement of the migrating parent(s) in their children’s lives (Danilewicz, 2010), on the family’s material situation (Walczak, 2016), and/or on their involvement in decision-making processes (e.g., Majerek, 2015). Some studies analyze the evaluation of transnational family life retrospectively (Danilewicz, 2021; see also Seidel, 2024 for Moldova). More fundamentally, however, is the lack of findings on how children—regardless of whether they have personal experience with it or not—think about this family arrangement, particularly against the backdrop of the pervasiveness of normative ideas of good childhood and family, as outlined above.

## 3 Study design

The following analysis is based on the sociological part of the larger bi-national and interdisciplinary research project DoDzi. The study aims to identify conjunctive knowledge about transnational families and good childhood. Group discussions with children were chosen as the instrument for data collection, based on two key considerations. Firstly, group discussions allowed us to identify the implicit, conjunctive knowledge (Bohnsack, 2012) of the participants. Secondly, compared to individual interviews, group discussions make it possible to (at least partly) distract from the power imbalance between the adult researchers and the child interviewees by focusing on interactions between the children (Vogl, 2005) and offering a feeling of safety and protection within the group. The groups were composed homogeneously regarding the children’s experiences with transnational family settings. The group categorization is methodologically relevant insofar as homogeneity in biographical experience is crucial for promoting a dynamic, participant-initiated discourse (Bohnsack, 2012) and a positive group atmosphere. To ascertain the children’s experience with transnational family life ahead of the group discussions, we distributed a short questionnaire at the schools, which the children completed on site. However, as our questionnaires and group discussions show, even families without migrating parents frequently have experience with such arrangements in their extended family circles. Thus, children of both groups—with and without direct experience of migration—were familiar with it to varying degrees. A comparison between those with and without migrating parents is not intended at this point.

As an introduction to the group discussions,^6^ the researcher read a fragment from the children’s book “Tata gotuj kisiel!” [Papa Makes Jelly; authors’ translation] by Stenka (2016). In the selected fragment, the mother, daughter, and grandmother discuss the mother’s plans to migrate temporarily to England. Nine-year-old Aśka, the book’s protagonist, first learns about these already solidified plans during the conversation. In her reaction, she shows that she disagrees with her mother’s decision and is outraged. During her mother’s three-month absence, Aśka is to live with her grandmother, and their apartment is to be sublet. The father is not mentioned in the passage. When selecting the fragment, we ensured that different perspectives on temporary migration were considered and that children were offered different points of connection, which they could then use to emphasize self-selected aspects from the story. After reading the passage aloud, the researcher first invited participants to recount its contents. The children were then asked to comment on the individual positions in the book and ultimately to talk about their own experiences. The children could choose for themselves to what extent they wished to contribute to the group discussion or to simply listen. In this way, they were offered a form of “exiting” the data collection situation (for “ethics in practice,” see Guillemin and Gillam, 2004; for a reflection on children’s “voice” and “silence,” see Spyrou, 2015).

All group discussions took place in schools. A total of seven schools were involved, with four schools from the western and three from the eastern parts of Poland. The questionnaires were distributed during personal visits to the schools by a native Polish-speaking researcher from the team, who was able to familiarize herself with the schools and obtain detailed information about the data collection. Information material for parents and children was written down and made available in the form of a video. Informed consent was obtained from parents and children on this basis. The data collection process was accompanied by repeated phases of reflection within the team (e.g., on the interviewer’s caution in asking too directly about experiences with parental migration; on dealing with the teachers). Care was taken to ensure that the teachers were not given access to the data collection or the collected data. Between June and December 2022, a total of 27 group discussions were conducted, in each of which three to five children between the ages of 12 and 14 took part. This article is based on 19 of these group discussions,^7^ which were fully transcribed and translated into German at the time of analysis. The analysis follows the guidelines of Strauss’ (1998) Grounded Theory approach. This means, for example, that the interpretation does not begin with a prefabricated category system, but that the categories and their connections are the result of an iterative-cyclical interpretation process. The generated readings are successively checked, modified, rejected, or (provisionally) confirmed through comparison with other cases. We followed Strauss and Corbin (1996, p. 63ff.) suggestion of contrastive comparison in order to increase theoretical sensitivity and augment the analysis. In this process, parental presence emerged as a central category: All interviewed children identify constant parental presence as an inherent expectation and relate this to the transnational family arrangement. Nevertheless, they do not follow a universalized idea of what children need or how families should look. Rather, they use different categories than those we are familiar with from adult discourses to evaluate this type of arrangement. Children’s contextualization of transnational families and their views on participation in the family strategy are the focus of the analysis in the following section.

## 4 Good childhood—transnational families from children’s perspectives

### 4.1 Parental presenteeism—a norm as a recurring benchmark

A relevant point of reference the children use to describe and evaluate the family presented in the book excerpt is the co-presence of family. At least in the beginning of the discussions, some groups evaluate the family presented in the book in the context of this shared understanding, thus identifying the presence of the mother as a natural expectation, in that the *“mother is also something like a part of the house”* (Leon, GD1). After the passage is read aloud, some children immediately adopt Aśka’s perspective and emphasize what children can reasonably expect from their parents:

*“It’s normal, for example, that she reacted like that, because I would react the same way in a situation like that. It’s understandable that she does not want her mother to leave.”* (Maja, GD2)

For many children, the thought of being separated from their mother for a longer period is initially almost inconceivable. Moreover, the physical co-presence of “both” parents is expected. In this way, the children quickly deviate from the book’s premise, in which the mother migrates and the father does not appear. In doing so, they also adopt a different perspective to that of the media discourse, which primarily problematizes the migrating mother. In the group discussions, the father is brought into the interpretations instead, upholding the ideal of co-present, heterosexual parenthood: *“It is probably the case that every child needs a father’s and mother’s hand”* (Max, GD3). Independent of gender, both parents are mentioned regarding the support they provide. The idea of a parent’s absence is partly fraught with fear, because:

*“A parent is basically our closest support. At least they should be the closest support. So if they were many kilometers away from us, it would simply be more difficult to get that support.”* (Alicja, GD4)

What emerges from the group discussions is, on the one hand, a conjunctive knowledge of the norm of the aforementioned physical co-presence of both parents. On the other hand, we also find an early distancing from this norm—sometimes formulated explicitly, other times rather subliminally. For example, in the quote above, Alicja does not question parental support per se, but rather considers it more difficult to realize from a distance (but even the “distance” is further differentiated, c.f. section 4.4). Such an “erosion” of the expectation of co-presence can be found in almost all passages that refer to this norm. In other words, absence does not automatically call good parenting into question, and migrating mothers are not (necessarily) devaluated. For instance, following Alicja’s statement as cited above, Maja also draws the conclusion that:

*“All in all, it’s not a bad thing, because she’s [the mother in the book] been offered a good deal and it’s kind of working out for her. But I would not want to be in that situation myself, for example.”* (GD2)

Staying with the norm of permanent co-presence for now, we can ask what is associated with it. Parents are described as children’s *“closest support”* (Alicja, GD4), guaranteed by their physical proximity. Co-presence ensures parents’ immediate availability if problems arise. As Marysia explains it: *“If something were to happen, [Aśka] might not feel that support from her mother because she would not be near her”* (GD5). In Marysia’s mind, physical proximity makes the support more tangible. Presence is not only required in the event of a crisis, but also for joyful events. In our group discussions, the children also express concern that absence could jeopardize the celebration of special events, such as birthdays they wish to spend together. In addition, the parental presence is also expected in daily life. Everyday interactions in a physically shared location allow space for children and parents to have spontaneous conversations, participate in each other’s lives, and foster closeness. Jonas is convinced that family life is more difficult at a distance:

*“because this person who goes abroad, one of the parents, is usually not up to date with these things […] and it’s also just that you do not want to talk about them over the phone, for example, and repeat everything that’s happened all day.”* (GD6)

In the children’s view, distance does not make closeness impossible, but it does create challenges and requires everyone involved to engage in new ways of “doing family” (Morgan, 1996). Recent studies worldwide show how actively children are involved in this, for example by maintaining contact (Nosek-Kozłowska, 2021; De los Reyes, 2023), controlling emotions toward parents (Gu, 2023), or conducting conversations based on their knowledge of which topics should be avoided over the phone (Hoang and Yeoh, 2014, p. 191). How children in our group discussions position themselves toward distance and closeness and how they imagine a good way of managing and shaping transnational family life is described in greater detail in section 4.4.

The absence of a parent can also mean that children must assume additional tasks previously performed by that parent. For example, Antia et al. (2020, p. 12) show that, “in the African and Eastern European regions, girls in migrant households tend to be more vulnerable than boys, regardless of which parent migrates,” precisely because they must take on more responsibility in the household and for their siblings. However, our analysis shows that children in Poland are primarily concerned about which adult will take on the care work, such as cooking or school drop-offs. Questions of daily routines, or even the wish that parents supervise children’s everyday lives, stand out: *“Yes, I would have a problem keeping myself organized because I have a bit of a problem managing my time”* (Jarek, GD7). As such, although the children repeatedly express concern as to who would assume their parents’ well-rehearsed routines, the generational order is not seen as fundamentally modifiable.

Physical co-presence is thus seen as a guarantee for an organized, protected, and anxiety-free childhood. Against this backdrop, temporary migration could have a *“very negative impact on the child […] because if one parent stays here and the other goes somewhere else, it also means that the child receives less attention”* (Zuzanna, GD8), thus risking the child’s central position in the family. A longer separation could *“maybe even [be] a bit damaging, maybe even traumatizing, that your mother left you here on purpose, for money”* (Ania, GD9). Being deliberately left behind, especially “for money,” contradicts the norm of a sheltered and child-centered childhood. In the latter half of the quotation, the norm of co-presence is highlighted but its relevance is nevertheless somewhat mitigated, insofar as the mother’s absence is not automatically associated with traumatization—instead, it could be either “a bit damaging” or, in extreme cases, “even traumatizing.” However, the deviation from the set norm is limited. Ania (GD9) continues: *“But on the other hand, she wanted to do something good for you.”* Thus, she does not question that the mother’s intentions (in the book) are in the best interest of her child. Some participants therefore reject Aśka’s indignation. Leon (GD1), for example, would *“not be the least bit indignant in Aśka’s place, because mother does it for the whole family, including me, to earn more money and improve our lives.”* A sheltered childhood can also align with the idea that parental migration can be the best decision for a child’s well-being, as there are *“times when they have to do something for the child’s welfare”* (Wiktor, GD10). The common denominator in the group discussions is that the child’s best interests must be the guiding principle of parental action. Permanent parental co-presence is an expected but not obligatory component of this.

Those children in the group discussions who do uphold the norm of co-presence largely agree that migrating parents (should) feel guilty, having failed to meet their children’s legitimate needs. While a few children emphasize the impossibility of making up for the loss of time spent together, some offer suggestions for compensation. For example, one discussant interprets the fictional mother’s plan to take a long-distance trip with her daughter after their separation as *“making up for the fact that she is leaving”* (Wiktoria, GD11). Others say the mother must “compensate” *“for leaving her, for doing something to her, not to upset her, but yes, something she did not necessarily want, or something like that, to apologize to her”* (Karol, GD2). Our findings from Poland thus differ significantly from those on transnational families in other countries. Particularly in Asian countries, children’s indebtedness to their parents for the sacrifices they make for their children by migrating for work is well documented (Madianou and Miller, 2012; Christ, 2017; Gu, 2022). In our group discussions, the participants also understand migrating parents to be acting in the interests of their children or family. Nevertheless, they view stayer children as the ones to make sacrifices, justifying any rebellion or demands for compensation. The children are blameless, while the parents must feel guilty for breaking with the norm of co-presence. They are expected to take all possible measures to make their absence as acceptable as possible for their children (c.f. section 4.4). Filial piety, children’s gratitude toward their parents, is not a guiding principle here.

The extent to which parental co-presence as a norm is perceived as effective and essential for the child’s well-being becomes clear in another way during the group discussions, namely when the participants discuss their own experiences with transnational family life. For example, Nadia (GD6) shares the story of how she and other family members promoted the father’s proposal to migrate temporarily to earn money as a confession: *“Honestly, we all say that’s good.”* Others explain that their peers do not necessarily know that their parents sometimes migrate. Just the idea of their knowing is:

*“[…] also a bit depressing because, for example, when your friends, I do not know, come home and there’s your mother and so on, and you are sitting with your grandmother, for example, well…then it’s a bit sad.”* (Natalia, GD3)

The mother’s absence is interpreted as a deviation from the normative, expected arrangement, which is “a bit depressing” precisely because of the comparison to others who meet this norm. Some participants even describe stigma management, for example by deciding whom they tell about their family life—and whom they do not. The impact of the norm of co-presence can also be observed when discussants describe their cautious interactions with classmates who (might) come from this type of family or from families living translocally for other reasons. Wiktoria (GD11) is unsure how to deal with such information about a classmate:

*“When I hear that someone is not living with their parents, I try not to act strangely, but I am sorry. Because I live with my parents, and if my parents had to move out, I would be very sad, so I am sad.”*

Here, too, are absent parents stigmatized and treated warily, almost as a taboo. Contrastively, some say they are envied for having a “free house”—that is, one free from parental control.

In summary, from the children’s perspective, parental co-presence is seen as an inherent part of the normative pattern of good childhood (see also Danilewicz, 2021, p. 173f.). At the same time, the group discussions reveal a differentiated approach to the phenomenon, far removed from the “moral panic” characteristic of adult discourses on “Euro-orphans.” Rather, the children erode the norm by identifying valid reasons for migration and exploring the options for designing an arrangement that would best suit them (c.f. section 4.4)—precisely because migration can be a vulnerable experience.

### 4.2 Valid reasons for deviating from the norm

*“How did Aśka react to that [mother’s migration]?”*

*Wiktor: “The way I think any child would probably react. She would not want to let go of her parents and not see them in person for three months.”*

*Kuba: “Because this child did not understand that you have to leave, that they just need this money.”*

*Wictor: “For example, she did not understand the situation the family was in.”* (GD10)

Even those children who adopt Aśka’s negative attitude at the beginning of the group discussion later name legitimate reasons for migration. Rather than categorically negating the possibility of a “good childhood” or “good parenting” despite the parents’ absence, they understand migration as a legitimate family strategy. Some children do not even interpret the book excerpt in the context of the norm of co-presence. Instead, they directly contextualize it for example in the current labor market. Alicja (GD4) summarizes the discussion of the opening stimulus as follows: It is about *“how the mother could not find work where she lived. She found a job somewhere abroad.”*

The discussants recognize that the mother tried to find a local solution to the problem of unemployment. Though she does follow the norm of a present mother, the context necessitates her legitimate deviation, locating the family as an economic unit in society. Following this line of reasoning, the family is not an island; rather, the members must react to social conditions, such as the poor labor market. Thus, families cannot always be structured the same way. Using Schütz (1932, p. 93ff.) terminology, one could say that the “because motives” are clearly developed. The children determine the reason(s) for the migration plan, for example the dismal job prospects in Poland. A distinction must be made between this and the “in-order-to” motives, which indicate how the action comes about and what the actor intends. The decision to migrate is considered legitimate (although not always desirable) by the children if it is made in the interests of the family: *“You have to think that it’s for the good of all of us, that they want money for us too”* (Artur, GD9). Hence, the legitimate reason to migrate is not only to secure their livelihood but also to strive for a “better life” by earning money abroad:

*“Why do you think Aśka, Aśka’s mom, decided to go abroad?”*

*Marysia: “Because she found a better job there where she could support everyone so that they could live better.”* (GD5)

Another discussant, Max (GD3), goes so far as to describe it as *“a sin*”—“it” referring not to the migration itself but to the mother’s turning down the lucrative offer of a three-month job. This employment could be an investment in their future. In short, families need not be in immediate hardship for children to recognize migration as a legitimate strategy. Instead, it is much more important that the child’s welfare is the recognizable guiding factor for decisions.

Unlike with the “Euro-orphan,” in which leaving for money is equated to abandonment, economy and emotion do not contradict each other here. Family is expected to be both an economic and an emotional unit. Thus, the assessment of migration is rarely unambiguous. Bartek (GD4) considers Aśka’s mother’s decision to migrate as *“good on the one hand, but also a bit…wrong on the other…”* while Max (GD3) argues that such a decision by the parents to migrate puts them in a *“kind of moral dilemma,”* as it involves ambivalent consequences.

Aside from the economic factors that legitimize migration as being in the family’s interest, the children identify further factors to consider when evaluating the family arrangement. Firstly, they refer to the quality of the child’s relationship to the migrating parent (for a more detailed discussion, see section 4.4). The discussants do not automatically assume that the relationship between parent and child is always good or that the distance is perceived as a loss. Secondly, they identify certain characteristics of the child to consider. Above all, they cite age as a decisive factor:

*“[…] but a child our age, for example, would not mind because they would know that Mom has to earn money, that they can get by with Grandma, that they will help Grandma, that Grandma will help them, at our age. And with a younger one, it would be that they cannot, that they would have to resign themselves to Mom going away.”* (Nadia, GD6)

Opinions vary as to the age at which an absent parent is problematic, but the dominant view is that the mother’s (and father’s) presence is particularly necessary for young children. A (young) child is depicted as being dependent. Within this context, the discussants also present themselves in the group discussions as actors who would cope well if their parents were absent. The interpretation that especially young children suffer from parental absence suggests that the discussants, who define themselves as adolescents, speak in an enlightened manner about migrating parents.

In summary: The children provide a range of valid reasons and conditions that legitimize the temporary migration of a parent. As Krystian (GD1) succinctly puts it: *“There are age limits where certain reasons are permissible. There are age and time limits. There are a lot of limits that make something acceptable or unacceptable to us.”* From the children’s perspective, parental migration therefore cannot be regarded categorically as a breach in “good childhood.”

### 4.3 Children’s role in the decision process and the time before parents depart

At the beginning of the group discussions, when the children review the book passage used as a stimulus, the mother’s decision to migrate is of central importance. However, the fact that the mother made the decision and that her daughter was not involved are problematized less than the point in time at which Aśka is informed of the said decision—namely, far too late:

*“But for Aśka it was also like…it was also a shock, because after all, the mother probably knew for a month, and [Aśka] only found out, I do not know, a week before she left.”* (Wiktoria, GD11)

According to the discussants, the “shock” for the protagonist is that she only learns of her mother’s migration plans last minute. The fact that Aśka is informed so late is repeatedly problematized in the group discussions; this seems to be a bigger grievance than the fact that she is not allowed to participate in the decision itself. This applies not only to the book family, but also to the participants’ own experiences:

*“For me, these first trips [of the father] were the most difficult […] I was angry with everyone else that nobody told me earlier, because I was younger at the time, so [the family] did not talk to me a bit, because it did not…it did not affect me then, and now somehow, when someone leaves, I know first because…well, I generally have better contact with my parents.”* (Mariusz, GD12)

In this quote, Mariusz voices his anger toward his family for not including him in the family affairs earlier. He explicitly ties this secrecy to his young age and the notion that his father’s migration would not “affect” him. However, he seems to have made his dissatisfaction clear to his family, as things have changed for the better: He is now the first to know when someone leaves, and he notes improved contact with his parents.

Secrecy toward children has been discussed and researched in various studies, for example among transnational families in Vietnam (Hoang and Yeoh, 2014) or Switzerland (Roulin and Jurt, 2014). Keeping migration plans secret from children is interpreted by adults as a form of protection, as children are not trusted to understand adult issues; parents also want to spare themselves and their children a long and difficult farewell (Roulin and Jurt, 2014, p. 203; Danilewicz, 2021). While the children interviewed in our study agree that keeping migration plans secret from children is unacceptable (and violates their sense of “doing family” well), they have differentiated views on when to involve children in the face of a possible or imminent (parental) migration:

*Wiktoria: “I would say, it if it’s already for sure and does not build up so much tension because it might not work out.”*

*Emilia: “But maybe, for example [the mother] could say it when they made the offer, for example. It’s not clear what the timing is.”* (GD11)

Wiktoria and Emilia discuss the pros and cons of different points in time and conditions they consider appropriate for informing the child: When the mother receives an offer, when she has accepted, or when it is certain that she will leave. According to the discussants, parents are required to balance children’s different needs: On the one hand, children should not be burdened with (unnecessary) tension; on the other, they have a legitimate right to be involved in the family’s affairs. However, the participants also consider the parents’ situation, such as the fact that they themselves sometimes find out about job opportunities abroad at very short notice. This example highlights the sensitivity that the discussants expect from parents to handle the situation appropriately and communicate the decision to their children at the right moment and in the right way.

The participants reflect in the group discussions why they consider it so important to know about the parents’ migration decision early on:

*“Well, but it’s logical that the child will not make that decision, but [the mother] should… She should take an interest in her opinion and not just ignore it.”* (Maciej, GD13)

Maciej’s use of the words “interest” and “ignore” make it clear what a significant difference it makes, from his perspective, whether the child’s opinion is heard. For Maciej, as for many other participants who formulate this demand similarly, listening is a question of the parents’ interest in the children—an interest that children expect. The children also emphasize the specificity of their perspective, in terms of their generational position and their individual experiences:

*“Because my parents, I mean, they sometimes cannot understand how I feel because they are adults, and in their case, for example, their parents never left. They also kind of do not know, kind of, how me and my siblings kind of feel about it, you know.”* (Matylda, GD12)

Matylda is referring to the challenge she and her siblings face in making their feelings about the separation accessible to their parents and thus engaging in a particular form of “emotional labor” (Mand, 2015, p. 26). Thereby, she addresses the fundamental problem of an intergenerational shift in perspective. According to her, what separates her and her siblings from their parents is not only the fact that they are adults, but also that the parents do not have their own transnational experiences as children from which to draw. Listening to children’s opinions is not merely a symbolic gesture of showing interest in the child. Rather, it creates a space and time for stayer children to be actively involved:

*“In my case, when my father left in September, it had already been planned somewhat since May. And we all talked about it together so that it wasn’t the case that I did not know and my father suddenly left. But we all talked about it around three months beforehand.”* (Mariusz, GD12)

Mariusz emphasizes the fact that the family discussed the upcoming change together at an early stage, and that the children were involved in the planning; he describes an interactive process that includes all family members. This can be interpreted as the children demanding a “doing family” in which their voices are heard, resonating, for example, with results from a study by Kutsar et al. (2014), which also pinpoints children’s wish to voice their opinions regarding migration-related decisions.

In contrast to the interactive process experienced and valued by Mariusz in the quote above, Natalia (GD3) describes a process taking place within herself:

*“I did not really have anything to say because my dad came back from work one day and said, announced, that he was leaving soon for three months. So I had…it’s nice that I had time to process everything in my head, not like from one day to the next, but I actually had a month, I think. And I got used to it and it did not hurt as much.”*

Although Natalia’s father merely “announced” the decision, she nevertheless emphasizes that it was “nice” to have time to “process everything in [her] head.” The prospect of separation, which Natalia experienced as painful, was at least mitigated by the time she was given to adjust. As in Matylda’s case mentioned above, Natalia also engages in a form of emotional labor by making herself adjust to a painful separation experience. Again, the period between being informed about the migration and the father’s actual departure is of central importance here. In the group discussions, participants name numerous processes that take place during this time: They grapple with the parents’ decision, sometimes protesting it or looking for alternative solutions, for example by suggesting strategies to find work closer to home. The children use this time to develop and express their own opinions, to imagine the time during the separation, and, if necessary, to plan for it. Children are therefore by no means passive, instead working actively to interpret the situation and position themselves within it.

### 4.4 Shaping and organizing the time of separation: children’s views on spatial, temporal, and relational aspects

It became clear in sections 4.2 and 4.3 that children do not categorically assess their parents’ decision to migrate as problematic, but rather differentiate regarding the legitimacy of migration reasons and the timing and type of communication about the decision. This also applies to the shaping and organizing of the period of separation, which our group discussion participants consider to be important for the evaluation of transnational family life. This subsection reconstructs, which notions of good childhood the participants apply with regard to the time of separation, and how normative expectations can be met—or modified. Numerous differentiations can be reconstructed from the group discussions in terms of the discussants’ perspectives on these questions. We have divided these differentiations into three central levels: the spatial-geographical, the temporal, and the relationship-interaction level.

The relevance of the “spatial-geographical level” concerns, for one, the distance between the parents’ place of migration and the children’s place of residence. For example, Oliwia mentions the need for parents to be able to quickly be with their children. The ability to bridge the gap, in the sense of an experienced or imagined closeness between the stayer child and the migrating parent, defuses the feeling of separation and inaccessibility. Against this background, Oliwia (GD12) rates her own transnational experience as positive—and better than a transnational experience between England and Poland, as in the book passage:

*“When my father went to Germany last year to work, yes, then from the beginning, it was about two weeks, it was long, despite everything he still came, you know? He could always get in the car and come, and here [referring to England] that’s not possible.”*

By emphasizing the possibility that her father was “always [able to] get in the car,” Oliwia affirms her father’s availability—and thus “repairs” the norm of parental presence. This aspect adds to the point mentioned in section 4.1, in which participants express their expectation for parents be close enough to respond quickly to emergencies. Here, the particularity of the European transnational space, which allows for the construction of such (geographical) proximity despite distance, becomes relevant for children. The “proximity” to parents’ place of work can also be produced through children’s own mobilities: *“When my father left, I only got used to it when I visited him together with my mother and brother”* (Oliwia, GD12).

Through her own experience of mobility to her father’s place of work, Oliwia felt closer to him, making it easier for her to accept and participate in transnational family life. While Oliwia does not elaborate on what exactly visiting her father achieved or changed, other participants talk about the “knowledge” they gain about their migrating parents’ faraway lives, giving them security and confidence:

*“And sure, I missed him a bit, but somehow not so much, because he just called me, we talked sometimes, I knew how he was. I know he went there just like that, almost like on vacation. So totally more on vacation. And I did not miss him so much because I also knew where he was going, I knew in advance and I knew what he was up to.”* (Alicja, GD4)

Alicja’s knowledge of her father’s location, condition, activities, and plans abroad quickly reduce the feeling of missing him and thus mitigates the norm of parental presence. This knowledge also reassures children that their parents are doing well, revealing another aspect of the normative pattern of good childhood: Children should not have to worry about their caretakers. Constructing parental closeness and acquiring knowledge about parents’ well-being are thus means by which children mitigate or repair the normativity of parental presence during their absence.

The spatial-geographical level encompasses not only the mobile parents’ place of migration but also the spatial changes for stayer children that may accompany this migration. This aspect has received attention in recent studies on transnational families, as the mobility experiences of stayer children (Mazzucato and Haagsman, 2022) can be considerable when having to move (usually within the country of origin), sometimes even several times. In our group discussions, the participants reflect on the implications that moving to another place or household can have for stayer children, such as in the case of the book’s protagonist: *“If she should go somewhere further away, to another city, let us say that she goes to study for that time. She would simply be cut off from her friends”* (Nadia, GD6). The participants consider how, if stayer children are physically separated from friends, they have to organize and maintain contact in other ways. The discussants recommend that adult caregivers actively support this:

*“As for this daughter’s grandmother, she could give her a few rules to follow, such as inviting a friend to stay with her for a while to make it a little better, to cover the bad with the good.”* (Helena, GD13)

Helena describes what can comfort the child during a separation, such as inviting friends to stay overnight. Helena, like other discussants, recognizes that transnational family life may also create challenges “beyond” the family, such as separations from significant others (e.g., friends). Along with parental absence, these disruptions contradict the normative pattern of a sheltered childhood. Nevertheless, the children point out ways in which such ruptures can be addressed to make the situation (more) acceptable. Also here, as with the distant parents, the study participants recommend creating closeness with the friends in new ways, by inviting them to visit or keeping in touch through (social) media.

In addition to the spatial-geographical level, the discussants also deem the “temporal level” significant. Relatively uniformly, longer separations are seen as more challenging and more consequential than shorter ones:

*“Well, if someone goes away for a month, that’s fine, a month is a short time, so you meet them again after a month and it’s normal. And if he goes away for six months, you do not really see him for six months. You miss him so much, you kind of do not have that…I do not know what to call it, but they are probably different feelings.”* (Natalia, GD3)

Natalia believes that, while it is easy to return to normal with the person after a short separation, it becomes more difficult with longer separations. Despite new technological means she is concerned that one cannot “really see” each other, and possibly develop “different feelings.” Although children consider the presence of the other person fundamental to experiencing connectedness, they nevertheless believe it is possible to spend a short time apart, whereby the classification of time periods as “short” varies. Constructing parental absence as “short” and thus as not damaging, in fact as causing no chance at all, is another way of coping with it and repairing the norm of parental presenteeism.

In addition to the question of duration, the group participants discuss the timing of parental migration and differentiate between various scenarios:

*“I think if they left during the school year, I would be fine for the first few days and then I would be tired because I would have to look after myself alone.”* (Dominik, GD7)

This example points to the importance that the study participants attach to the everyday—and normative—obligations of stayer children, for example as school children, and to their temporal structure. Dominik assumes that he would be “tired” of looking after himself after just a few days, presenting parental support to be necessary during the school year. It is considered less necessary during vacation: *“It was summer vacation at the time, so it was okay”* (Adrian, GD3). This corresponds to children’s expectations that their parents organize and supervise their lives, as discussed in section 4.1 (p.11). Expectations from parents, as well as the demands children place on themselves (being disciplined, getting good grades, planning time for homework and extracurricular activities, etc.), illustrate how demanding being a schoolchild is. The following quote shows that these demands must also be met during parental absence:

*“Was there anything difficult when your parents were not there for you?”*

*Natalia: “Hmm…Not really, because back then I did not have chemistry or physics, and it wasn’t as difficult as it is now in seventh grade, it was easier in fourth grade, and I had to learn some simpler things. And my grandparents are into history and things like that, they know it very well.”* (Natalia, GD2)

Natalia assesses whether parental absence caused difficulties based on whether it led to problems at school or whether the temporary carers, in this case her grandparents, were able to provide the necessary support. From the children’s perspectives, whether a transnational family arrangement is acceptable is also measured by whether stayer children can fulfill their responsibilities as (good) schoolchildren. The norm of good childhood can thus be met, despite parents’ absence, when being a good schoolchild remains possible and when parental support is substituted by the (temporary) carers.

The quotes above already suggest that, in addition to the spatial-geographical and temporal levels, the participants also consider the “relationship-interaction level” to be significant for shaping the separation period, especially on an emotional level. This becomes evident, for example, when children mention worries that contact with migrating parents may suffer, as in the case of the book:

*“And let us assume that [Aśka] then gets an SMS from her mother saying that she can only call in three days because she has some kind of situation there, she has to sacrifice a lot of time, then for the child that’s like…The child might have the feeling that she is simply being rejected by her parents.”* (Wiktor, GD10)

Like Wiktor, many children in our group discussions express the fear that relationships with migrating parents could deteriorate as a result of less intensive and intimate contact, and they anticipate the additional effort that would have to be invested into rebuilding the relationships after the parents’ return: *“So later on, when she arrives, that relationship has to be rebuilt because she did not know what was going on before, a lot may have changed in that time”* (Maciej, GD13).

On the other hand, stayer children’s (positive) relationships with their migrating parents are considered a support for upholding an intact family life and a protected childhood. This may be the case when families find effective ways of maintaining contact and intimacy despite the distance, as Robert (GD12) reports from his own experience:

*“So for me it’s actually not long, my parents often come, you can visit them, or you can talk via various apps, like Skype and so on. […] My father called my mother every evening and we told each other what we had been doing all day.”*

Robert emphasizes the mutuality of both his own and his parents’ efforts to maintain a close connection, including reciprocal visits, regular communication, and the readiness to share what is going on in each other’s lives.

The group discussants’ accounts pinpoint the centrality of stayer children’s relationships to their migrating parents (and not, in general, of the parent’s gender) when evaluating transnational families. They focus not only on the question of presence or absence but also on complex processes in which they factor in the individual history of a relationship, the constraints faced by both parents and children, the type of contact they are able to maintain, and the (anticipated) possibility of processing the separation phase, in the sense of “rebuilding” the relationship. Children present parental relationships as dynamic and flexible and develop solutions to maintain a sense of connectedness with one another despite the distance. In this way, children construct transnational families as adaptable and thus as able to meet children’s needs of close and emotional relationships, at least when parents (and children) invest the necessary efforts.

Equally relevant are the relationships with those adults who remain with stayer children. These include the parent who stays behind or other caretakers such as grandparents. In the group discussions, the participants reflect on how they feel about various relatives, what kind of relationships they have with them, and what advantages or disadvantages there are or would be to living with them. Ania and Szymon (GD9) imagine themselves in the role of the book protagonist, who will be staying with her grandmother:

*Ania: “It depends on who I would stay with. I mean, if I lived with my grandmother, well, that would be mediocre, because let us not kid ourselves, my grandmother is like, hmm. Anyway, my grandmother lives in the countryside, so I’d be even more….”*

*Szymon: “There’s no internet.”*

*Ania: “There is. But in general, well, in general, I do not know, for me on the one hand my grandmother, well, that might actually be pretty cool.”*

Ania and Szymon’s conversation represents many of the considerations expressed by discussants when assessing (hypothetically) how and where to live with other caretakers during parental absence: Whether in the countryside or in the city, with or without internet, whether the caregiver understands teenage concerns, whether they are too strict or too lenient, etc. These aspects and more are considered by the discussants when evaluating whether a care arrangement contributes to what can be called “adequate presence”—emphasizing the surrounding in which stayer children remain, and complementing their ways of handling parental absence.

Based on their own transnational experiences, participants refer especially to the changes in their everyday life that come from sharing it with other people and having to reorganize it while parents are absent:

*Natalia: “Then also when they come back later, it’s so strange because after two days when my grandmother was with me, for example, she had a different way of living with me day by day and my parents had a different way of living with me and it becomes a bit like that…”*

*Maja: “All in all, that’s true, too.”*

*Natalia: “You live differently with different people.”* (GD2)

Living in new care arrangements is accompanied by changes in everyday life to which everyone involved must adapt. This is true not only for the time of separation but also when parents return, requiring children to re-adapt. In the group discussions, this is discussed as a certain imposition that children must bear. Thus, it can be inferred that adults are inherently expected to provide a childhood that keeps children free from this kind of pressure to adapt. Children consider this the parents’ duty to make up for their “guilt” of leaving the children (c.f. section 4.1, p. 8). Adults are thus expected to acknowledge and support children’s “adaptative work.” Part of supporting stayer children with that adaptive work is to prepare and facilitate their transitions to new care arrangements, such as in the book family’s case:

*Emilia: “For example, the mother could take her to visit her grandmother more often, then maybe she would get used to living with her grandmother.”*

*Wiktoria: “And then: ‘Oh, grandma, did you buy a little dog?’ I’m not kidding, the grandma could have bought a puppy.”* (GD11).

The girls suggest that the mother initiate intensive relationship work between the stayer child and the new caretaker so that the former can adapt to living with the latter and the relationship and trust between them deepens. Interestingly, Wiktoria envisions that the mother takes the child to the grandmother’s and the grandmother’s buying a puppy, explicitly assigning the responsibility for the adaptive support and emotional well-being of the child to the adults. From the discussants’ point of view, such intensive relationship work should be aimed at establishing intimacy and supporting the stayer child’s transition to the new care setting. Again, this adds to the notion of adequate presence and serves to maintain a protected childhood in which competent and emotionally responsive adults are held responsible for the welfare and well-being of the (vulnerable) children.

To summarize, our study participants evaluate the shape and organization of the separation period using diverse aspects of the spatial-geographical, temporal, and relationship-interactional level. Importantly, the children consider the question not only of diminishing the worries connected to parental absence (by establishing closeness through regular communication and possibly visits, substituting for school-related support etc.) but also of adequate presence: The quality of relationships with new carers and appropriateness of new living arrangements form the basis for evaluating the transnational family. However, children also look beyond the family; not only relationships with parents and new carers, but also with friends should be ensured for the time of separation. Children demand to be involved in the planning and development of new care- and living arrangements during parental absence, while simultaneously expressing the expectation that adults (especially the parents) ensure that children’s needs are fulfilled at all times.

## 5 Discussion

In newspapers, television reports, and children’s books in Poland, transnational families, and particularly (temporarily) migrating mothers, are repeatedly portrayed as deviating from “good parenting” or “good motherhood” by defying the normative ideal of present parents. The weighted term “Euro-orphans” interprets this family form as one that neglects children’s innate needs, stripping it of any legitimacy. However, these are concerns voiced mainly by adults. Based on group discussions with children in Poland, we shift this perspective and ask children how they themselves view and evaluate this family arrangement, and we analyze which norms of good parenting can be identified when they discuss transnational families. The article thus goes beyond the specific subject of transnational families and captures the normative pattern of good childhood and parenthood from the perspective of children, which is closely linked to their experiences of being children in Poland. This childhood is characterized by children’s demands to be listened to, echoing a historical, albeit variable and non-linear, shift of (intergenerational) family interactions toward a more balanced, negotiation-oriented style, which has been described for several European post-war societies (for the Netherlands and Germany: du Bois-Reymond, 1994; for Poland: Adamski, 2002; Tyszka, 2004).

For the participants of our study, their demand for inclusion in family negotiations, in this case on parents’ mobilities, does not mean that they demand the same position as their parents in the family hierarchy. On the contrary, children refer to their vulnerability quite explicitly by expressing their dependency and their need for responsible adults’ care, control, and emotional comfort. Interestingly, the participants of our study offer a different interpretation to those offered in adult discourses: Instead of concluding that children should not be left behind by migrating parents in the first place (as frequently voiced in adult discourses), our discussants request that children be included in the shaping of an appropriate (temporary) substitute care-arrangement—to protect them from the vulnerabilities specific to their subaltern position in the generational order. The children’s notion of a “good family” that can be reconstructed from our group discussions is that of a child-centered family in which children are listened to and involved in the family’s decisions, so that they can view migration as a family strategy. Both parental presence and absence are measured against this backdrop. We will discuss our findings now through this lens.

At the beginning of the group discussions, directly following the book stimulus, the children refer to parental presenteeism as a norm. For them, as for the book’s protagonist, permanent parental presence is a fundamental and shared expectation. Already, the first variance to the media discourse on “Euro-orphans” emerges, insofar as the children also include the father in the family, instead of critically discussing only the mother’s absence—as is the case in our stimulus.

Further, the analysis shows that the norm of parental presenteeism erodes during the discussions, even for those children who initially supported it strongly. Overall, it is striking that the children do not argue based on universal children’s needs, but (also) conceptualize the family as an economic and socially contextualized unit whose actions can be evaluated respective to the specific situation. For example, the state of the labor market can legitimize temporary migration, as it enables a “better life” for the family or/and the children.

Although some children emphasize being happy that both their parents work in the same place the children live, the group discussants are far from scandalizing parental migration categorically. They discuss to what extent migration can be a threatening experience, but they do not reject outright the transnational arrangement itself. Rather, they problematize associated implications, an element missing from adult discourses.

It is less parents’ migration decisions that children criticize, but rather the fact that they are not involved in or informed of the decision-making process. The children’s demand to be informed at an early stage can also be interpreted against the background of Strauss’ (1974) reflections on how people deal with turning points—as the absence of a parent can be understood. Strauss aptly formulates how turning points challenge the self. He gives the example of “betrayal,” in the sense of an event occurring that the individual did not anticipate. With adults in mind, Strauss (1974, p. 106) writes that “an essential aspect of this critical experience is that the self-assessment of the person concerned is deprived of orientation.” But children who are confronted with the planned departure of their parent(s) at short notice also experience their self-assessment as sheltered children in the family and their routines being called into question. They demand to be given time to adjust to this (more or less relevant) turning point and to redesign themselves to some extent. This also includes helping to shape the new arrangement.

This demand by children to be informed early on goes hand in hand with a second requirement: The evaluation of the transnational family arrangement is based not only on parent(s)’ absence but especially on the presence of a (new) caregiver. The children expect a say in when, where, and with whom they will stay. These questions are particularly central for shaping and organizing the separation period and demonstrate a further departure from a universal conception of family and child, instead considering differing contexts, outlined above using the three levels, namely the spatial-geographical, the temporal, and the relationship-interaction level. In addition to focusing on new caregivers, the children also look beyond the family by emphasizing the importance of maintaining friendships and fulfilling their roles as schoolchildren; these can be reconstructed as further aspects the children perceive as “adequate presence.”

The main objective of this article is to identify how children evaluate transnational family arrangements, triggered by the “moral panic” featured in the adult discourses dominating public media. Our study indicates that this moral panic exhibits an adult bias. This does not mean that children do not problematize the family arrangement. Rather, they (also) problematize aspects that adults do not address, which can be related to their specific position in the social and generational order and offer a different perspective on the social world than that of adults. It is apparent that children construct a specific idea of good childhood that predominantly emphasizes space for them to shape their family as well as individual lives, extending the view beyond the intra-family sphere to include, for example, their roles as friends and schoolchildren.

This aspect in particular offers potential starting points for further studies. Existing studies on doing family over distance, important and widespread in research on transnational families, reveal how children contribute to the shaping of family, yet there is a notable lack of reflection on children’s position in the family structure. It is precisely this element children deem relevant when assessing the quality of transnational childhood. Furthermore, the results of this study encourage us to consider children as more than merely family members, insofar as the transnational family arrangement also has ramifications for relationships outside the family.

Furthermore, the group discussions clearly indicate the need to expand research on transnational families on those in Europe. For example, the generational structure in Poland is not built on filial piety, as seen in studies on transnational families in the Philippines. While children there are obligated to demonstrate gratitude toward their parents, some of the children we interviewed formulate an intrinsic demand that parents “make up for” their absence. In contrast, they hardly worry about taking on more responsibilities at home when their parents migrate, instead expecting to be well looked after. These points, which are closely tied to the specific generational (and gender) structure, are rarely found in societies with other generational (and gender) structures. The children in our group discussions refer to specificities of European transnational spaces, including geographical proximity when assessing transnational arrangements. Moreover, labor migration within the EU has become a common and familiar family strategy—everyone has someone in the family who works in another European country. Children increasingly grow up in this transnational reality and our study shows that they on the one hand reproduce the norm of a good childhood, but on the other also erode it, giving insight into children’s agency in a process of re-interpreting society.

## Data Availability

The original contributions presented in the study are included in the article/supplementary material, further inquiries can be directed to the corresponding author.

## Footnotes

^1^The acronym is derived from the Polish title "Dorastanie w rodzinach transnarodowych. ‘Dobre dzieciństwo’ z perspektywy dzieci" (DFG: 465048370). Alexandra König leads the German side of the project and Dorota Michułka the Polish side, which examines from the perspective of literature studies and reception research the way in which children’s books about transnational families are experienced and received. You can find more information about the project at "http://www.uni-due.de/biwi/koenig/dodzi/".

^2^To our knowledge, current statistics on temporary parental migration are not available (see also: Doiczman, 2017, p. 316). Further findings from the 2022 census in Poland have yet to be published.

^3^Studies on other Eastern European countries show similar findings (see Juozeliūnienė and Budginaitė (2018) for Lithuania, Seidel (2022) for Moldova, and Ducu (2013) for Romania). Instead of “Euro-orphans,” terms are used such as “abandoned children” in Romania, “social orphans” in Ukraine, and “white orphans” in Moldova (Lutz, 2018, p. 69).

^4^For example, a study from Poland shows positive effects on the willpower and solution orientation of children living transnationally (Doiczman, 2018), while Sordyl-Lipnicka (2020, p. 234) identifies the highest anxiety levels in children whose mothers or both parents migrate temporarily.

^5^The particularities of Polish migration are outlined similarly in the proposal of our follow-up project "(Self-)Positioning in transnational spaces – Children’s narrations and narrations about children living transnationally" (TraNa) which is funded by the DFG/NCN (GEPRIS 465048370).

^6^For more detailed information on the implementation of the group discussions and our own reflections, such as on generational power relations and the bi-national and multilingual research project, see also Schwittek and Jendrzey (in press).

^7^A table with characterizations of the 19 analyzed group discussions can be found in the Appendix Table 1.

## Appendix

**APPENDIX TABLE 1:** Description und characteristics of the group discussion based on the questionnaire.

Name of the group discussion	Group characteristics: children’s experience with migrating parents and geographic area
GD1	Children without experience of migrating parent(s); urban area; western Poland
GD2	Children with experience of migrating parent(s); urban area; eastern Poland
GD3	Children with experience of migrating parent(s); urban area; eastern Poland
GD4	Children without experience of migrating parent(s); urban area; eastern Poland
GD5	Children without experience of migrating parent(s); rural area; western Poland
GD6	Children with experience of migrating parent(s); rural area; eastern Poland
GD7	Children with experience of migrating parent(s); urban area; western Poland
GD8	Children without experience of migrating parent(s); rural area; western Poland
GD9	Children without experience of migrating parent(s); urban area; eastern Poland
GD10	Children without experience of migrating parent(s); urban area; western Poland
GD11	Children with experience of migrating parent(s); urban area; eastern Poland
GD12	Children with experience of migrating parent(s); urban area; western Poland
GD13	Children without experience of migrating parent(s); urban area; eastern Poland
GD14	Children with experience of migrating parent(s); rural area; western Poland
GD15	Children with experience of migrating parent(s); rural area; western Poland
GD16	Children without experience of migrating parent(s); rural area; western Poland
GD17	Children without experience of migrating parent(s); rural area; western Poland
GD18	Children with experience of migrating parent(s); rural area; eastern Poland
GD19	Children with experience of migrating parent(s); rural area; eastern Poland
